# Evaluation of the Water-Blocking Performance of Polyurethane Plugging System and Urea-Formaldehyde Plugging System in Xinjiang Oilfield

**DOI:** 10.3390/gels12060469

**Published:** 2026-05-28

**Authors:** Qianbing Lin, Guanyu Chen, Ruiqiang Dong, Xinyue Cui, Shiyu Zhang, Daoyong Li, Xinzhe Li, Lianghui Guo

**Affiliations:** 1 School of Engineering, China University of Petroleum-Beijing at Karamay, Karamay 834000, China2025015993@st.cupk.edu.cn (S.Z.);; 2 Xinjiang Key Laboratory of Multi-Medium Pipeline Safety Transportation, Karamay 834000, China

**Keywords:** polyurethane plugging system, urea-formaldehyde plugging system, low-permeability reservoir, water-blocking, plugging performance

## Abstract

Water channeling during water injection in mid-to-late development stages of low-permeability reservoirs in Xinjiang leads to a rapid water cut increase and reduced oil displacement efficiency, causing significant economic losses. This study systematically investigated two chemical systems—a polyurethane (PU) plugging system and a urea-formaldehyde (UF) plugging system—under simulated Xinjiang reservoir conditions. The PU plugging system formulation was optimized through control variable experiments, and both systems were evaluated for rheological properties, curing behavior, mechanical strength, salinity and temperature adaptability, and plugging performance using sand-pack displacement tests. The optimized PU plugging system cured at 80 °C for 1 h achieved a maximum plugging rate of 96% and a breakthrough pressure gradient of 4.2 MPa·m^−1^. The UF plugging system exhibited distinct temperature-triggered gelation, with viscosity rising exponentially above 60 °C, providing a plugging rate of 70% and a breakthrough pressure gradient of 1.0–2.0 MPa·m^−1^, suitable for deep fluid diversion. The PU plugging system offers high-strength near-wellbore plugging, while the UF plugging system enables controllable deep fluid diversion. Their complementary properties provide a comprehensive technical strategy for water channeling control in different low-permeability and high-temperature reservoirs in Xinjiang Oilfield.

## 1. Introduction

Low-permeability reservoirs in Xinjiang, northwest China, represent a critical strategic area for enhancing domestic oil and gas production [1]. These reservoirs are characterized by substantial hydrocarbon reserves locked within complex geological formations with poor physical properties, including low porosity, fine pore throats, and strong heterogeneity. The inherent challenge in developing such reservoirs stems from their insufficient natural energy, which often leads to rapid production decline and poor recovery efficiency during mid-to-late development stages. To address this, pressure-assisted water injection technology has been widely implemented across Xinjiang Oilfield. This technique involves injecting water at high pressures to replenish formation energy, create extensive micro-fracture networks that reduce oil-water seepage resistance, and utilize the differential flow characteristics between oil and water phases to mobilize remaining oil [2]. While theoretically sound, the practical application of this technology in Xinjiang’s specific reservoir conditions faces significant and persistent challenges [3].

The dominant issue arises from the strong reservoir heterogeneity and the presence of dense, interconnected natural fracture systems. During pressure-assisted injection, injected water tends to preferentially channel through these high-permeability pathways in a “short-circuiting” manner, completely bypassing the oil-rich low-permeability zones. This phenomenon leads to premature water breakthrough in production wells, characterized by a sharp increase in water cut and a corresponding dramatic decline in oil production rates [4,5]. According to the 2024 development annual report from Xinjiang Oilfield, this channeling problem has resulted in annual economic losses exceeding 1 billion RMB. The effectiveness of pressure-assisted injection is further undermined by remarkably short water breakthrough cycles, often less than three months after treatment for individual wells, with ineffective water injection accounting for up to 35% of the total volume [6]. This situation poses a major obstacle to improving the economic efficiency and sustainable development of Xinjiang Oilfield, necessitating the development of more effective water control technologies tailored to these challenging conditions.

Conventional channeling control technologies have demonstrated limited effectiveness when applied to Xinjiang’s challenging low-permeability reservoirs. Cement-based plugging agents, including water-based varieties with large particle diameters, are primarily suitable for plugging high-permeability formations and cannot effectively match the fine pore throats characteristic of Xinjiang’s low-permeability strata [7]. While ultra-fine cement offers better size compatibility, its practical application is constrained by rapid hydration rates, short initial setting times, and significant safety concerns, particularly in deep, high-temperature wells [8].

Particulate plugging agents, which rely primarily on mechanical blockage mechanisms, face challenges related to suspension stability with larger particle sizes, frequently resulting in inadequate plugging strength, inconsistent performance, and short functional longevity in field application [9]. Traditional gel systems, meanwhile, suffer from insufficient tolerance to the high temperatures and high mineralization degree encountered in Xinjiang reservoirs, coupled with difficulties in controlling gelation kinetics precisely, making them unreliable for Xinjiang’s demanding reservoir conditions [10].

**Figure 1:**
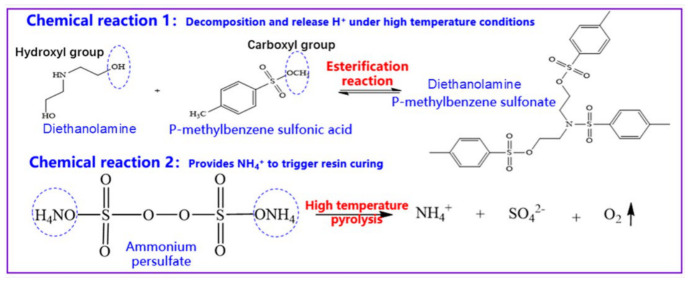
Reaction equation of the latent curing agent control mechanism [17] (adapted with permission from Shuanggui Li, Biao Qi, Qitao Zhang and Jingbin Yang, Gels; published by MDPI, 2024).

Following a background investigation into the gelation of resin-based materials, we found that in recent years, resin-based chemical systems have emerged as promising alternatives for advanced plugging applications [11,12]. They exhibit excellent heat and salt resistance. When injected into the formation, they have low viscosity. Upon heating, oligomeric resins and curing agents form a dense three-dimensional cross-linked network structure with a breakthrough pressure gradient of 4.2 MPa·m^−1^ and plugging rate of 96%, which is typically used to plug wider fractures in deep high-temperature oil reservoirs. Once formed, such resins are difficult to dissolve in the aqueous phase but are more easily soluble in the oil phase or acid solution [13]. After being injected into the wellbore, polyurethane resin undergoes cross-linking reactions initiated by a catalyst under specific temperature and pressure conditions, forming a high-strength plugging layer. Xiong et al. [14] successfully applied polyester temporary plugging particles to deep reservoirs in the Tarim Basin of China at 170 °C, achieving a 95.6% construction efficiency. After the operation, the average single-well production increased by 3.5 times. Chen et al. [15] developed a low-viscosity epoxy resin plugging agent that maintains a plugging strength exceeding 10 MPa after 24 h of aging at 140 °C, demonstrating excellent stability. After 160 h of aging, it can completely degrade into a solution, resulting in low reservoir damage. As shown in Figure 1, Yang et al. prepared a low-viscosity resin system with a temperature resistance of 140 °C and a plugging capacity of up to 13.07 MPa. The degradation rate of the system after dissolution can reach 97.69%, effectively meeting the plugging requirements of Tahe Oilfield. Meanwhile, before adding the curing agent, the curable resin remains in a low-viscosity, flowable state. After curing, it develops a certain degree of strength. For example, urea-formaldehyde resin undergoes a chemical reaction with the curing agent, gradually curing into a glue that forms a stable plugging structure [16].

Among resin-based materials, PU plugging system materials have attracted considerable attention due to their exceptional mechanical properties, remarkable resistance to saline environments, and the ability to form stable, robust cross-linked networks within formation structures [18]. The molecular structure of PU plugging systems can be strategically designed and optimized through selective formulation, potentially allowing them to meet the specific challenges presented by complex reservoirs in Xinjiang. Similarly, UF plugging system resins have shown potential as flow regulators due to their controllable gelation behavior and good chemical stability under reservoir conditions [19]. However, systematic comparative studies evaluating the adaptability and complementary application of these two systems under Xinjiang’s specific reservoir conditions remain scarce in the literature.

This study optimizes both polyurethane and UF plugging systems for low-permeability, high-temperature, and high-mineralization degree reservoirs in Xinjiang Oilfield. (This paper selects the J6 Area of the Karamay Oilfield as the subject of study.) The specific formation parameters are as follows: Formation temperature is 18 °C; Reservoir temperature after experimental/steam flooding is 80 °C; Total mineralization is 5113.96 mg/L. We systematically compared the performance indicators of PU and UF plugging systems. Through comprehensive laboratory evaluations of gelation mechanisms, rheological behavior, compressive strength, and plugging performance, we demonstrate that the PU plugging system provides high-strength near-wellbore plugging, while the UF plugging system enables controllable deep fluid diversion with distinct temperature-triggered gelation above 60 °C. The complementary properties of these two systems offer a comprehensive technical strategy for water channeling control in heterogeneous low-permeability reservoirs. The findings provide reliable technical support for improving oil recovery in Xinjiang Oilfield and similar challenging reservoirs worldwide.

## 2. Results and Discussion

### 2.1. FTIR Image

**Figure 2:**
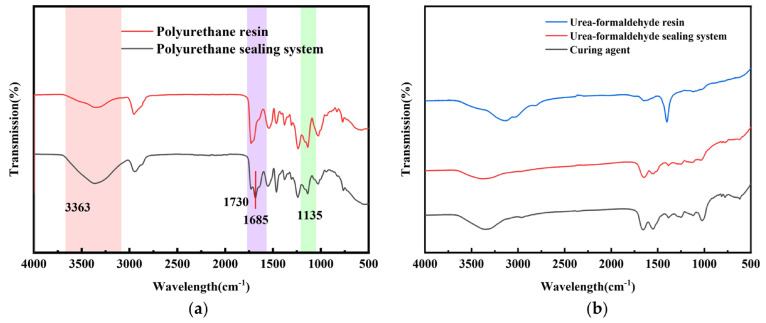
Infrared spectra system before and after curing. (**a**) PU plugging system; (**b**) UF plugging system.

As shown in Figure 2a, the infrared spectra of the PU plugging system before and after curing, it can be observed that a strong characteristic absorption peak of isocyanate groups exists near 2270 cm^−1^ before curing. After curing, this characteristic peak completely disappears. Meanwhile, a broad stretching vibration peak of hydrogen-bonded hydroxyl and amino groups appears at 3363 cm^−1^, a characteristic absorption peak of the carbonyl in urethane bonds emerges at 1730 cm^−1^, and a stretching vibration peak of is present at 1135 cm^−1^. The changes in these characteristic peaks directly confirm the consumption of isocyanate groups and the formation of crosslinking bonds, indicating that the PU plugging system has completed crosslinking and gelation. In the infrared spectroscopy test, after curing of the UF plugging system, the characteristic absorption peak of the amide carbonyl at 1685 cm^−1^ showed a significant increase in intensity, as shown in Figure 2b. The N-H stretching vibration peak in the range of 3200~3400 cm^−1^ became narrower, and its intensity decreased. Meanwhile, a characteristic absorption peak of the methylene ether bond appeared near 1050 cm^−1^, confirming the occurrence of condensation polymerization and the formation of a cross-linked structure.

### 2.2. Gelation Time of the Gel System

The gelation time data for polyurethane and UF plugging systems under different resin and curing agent concentrations are presented in Table 1 and Table 2. From these tables, it can be seen that under unchanged other conditions, the gelation time of PU and UF resins exhibits different variation patterns. For PU, when the curing agent concentration varies within the range of 0.5% to 3%, it has no significant effect on the gelation time, which is mainly determined by the resin concentration: at a resin concentration of 20%, the gelation time remains stable at 4.5 h; when the resin concentration is 30% or 40%, the gelation time shortens to 1 h; and when the resin concentration is 50% or 60%, the gelation time increases by 0.5 h compared to the previous values. For UF resin, an increase in resin concentration significantly shortens the gelation time, while an increase in curing agent concentration within the range of 2% to 6% only has a slight effect on the low-resin-concentration group: at a resin concentration of 20%, as the curing agent concentration rises from 2% to 3%, the gelation time decreases from 3.5 h to 3 h; however, no effective data supports higher curing agent concentrations due to limited solubility of UF. At resin concentrations of 50% or 60%, the gelation time is unaffected by changes in curing agent concentration and remains stable at 1.5 h. In comparison, the gelation time of PU is more sensitive to changes in resin concentration, with a noticeable shortening in the 30% to 40% range. Meanwhile, UF generally has a shorter gelation time and can stabilize at 1.5 h under high resin concentrations, demonstrating a faster gelation rate.

**Figure 3:**
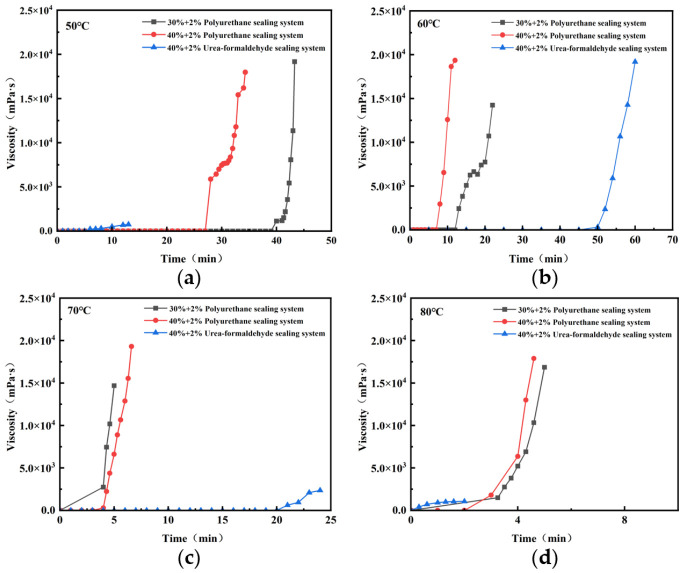
Viscosity of PU and UF plugging systems at different temperatures. (**a**) 50 °C; (**b**) 60 °C; (**c**) 70 °C; (**d**) 80 °C.

To quantify the gelation mechanics, we defined the gelation time as the time required for the system viscosity to reach 10,000 mPa·s under isothermal conditions. Figure 3 shows the viscosity evolution over time for both systems at different temperatures. For the PU plugging system, the viscosity increases slowly at first, then sharply after a certain induction period. The higher the temperature, the earlier the viscosity surge occurs. At 80 °C, the 30% + 2% formulation reaches 10,000 mPa·s in approximately 18 min, while the 40% + 2% formulation reaches it in about 12 min, indicating that higher resin concentration accelerates gelation. For the UF plugging system, the viscosity also increases with time, but the gelation is more gradual. At 80 °C, it reaches 10,000 mPa·s in about 35 min. At 50 °C, the viscosity remains below 10,000 for over 2 h, showing that temperature is a critical trigger. Based on the 10,000 mPa·s criterion, the gelation times for all tested formulations were calculated and are summarized in Table 3. In general, polyurethane gels faster than urea-formaldehyde under the same temperature, and both systems exhibit accelerated gelation with increasing temperature.

**Figure 4:**
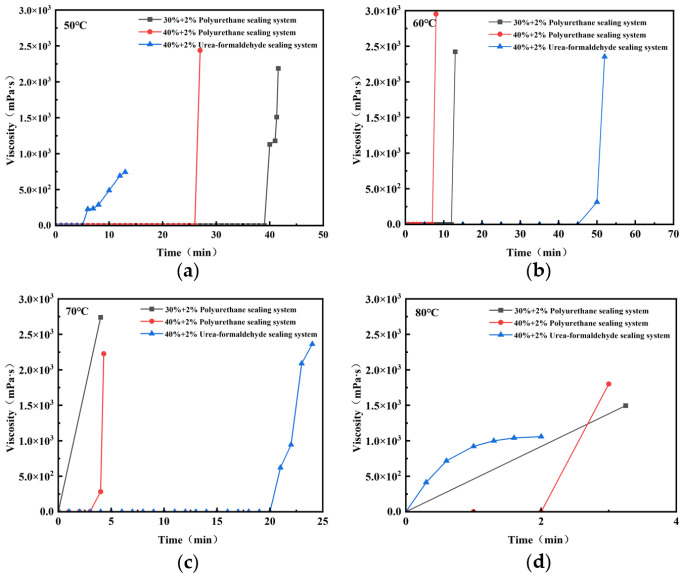
Viscosity pumpability windows of PU and UF plugging systems at different temperatures. (**a**) 50 °C; (**b**) 60 °C; (**c**) 70 °C; (**d**) 80 °C.

To demonstrate the effects of different operations, we define the ‘pumpability window’ as the period during which the system viscosity remains below 2500 mPa·s (the practical upper limit of conventional injection pumps). As shown in Figure 4d, at 80 °C, the 40% + 2% PU plugging system exhibits a pumpability window of less than 4 min, after which the viscosity rapidly exceeds 10,000 mPa·s. This rapid transition renders it highly suitable for near-wellbore plugging, as the quick gelation prevents flushing. In contrast, Figure 4b indicates that the 40% + 2% UF plugging system maintains a pumpability window of 10 min at 60 °C and over 30 min at 50 °C. The results indicate that the viscosity of the high-strength curable resin system remains stable before curing as the shear rate increases [20]. This extended low-viscosity period allows for deeper penetration into the reservoir prior to temperature-triggered gelation, making it applicable for deep fluid diversion and profile control.

The temperature-dependent pumping window of the UF plugging system exhibits a high degree of compatibility with the actual temperature gradient of oil reservoirs in the Junggar Basin, Xinjiang. A large volume of field measurement data indicates that the present-day average geothermal gradient in the Junggar Basin is 21 °C/km [21], characteristic of a low-geothermal-gradient basin. Long-term injection of ambient surface water into water injection wells has created a distinct low-temperature cooling zone near the wellbore, where temperatures generally remain below 60 °C. As the system migrates deeper into the formation, temperature increases steadily with depth and reaches 60 °C near a depth of 2000 m. This natural temperature distribution aligns closely with the 60 °C gelation trigger threshold of the UF plugging system: the near-well low-temperature zone ensures the pumping window and deep migration of the system; the deeper high-temperature zone triggers rapid gelation to achieve precise plugging. 60 °C serves as both the boundary temperature between the ‘near-well cooling zone’ and the ‘original formation temperature zone’ in this area, and as the critical temperature for the urea-formaldehyde system transitioning from an injectable state to a plugging state [22]. This threshold characteristic enables the UF plugging system to achieve targeted regulation of ‘injectability near the well and plugging in the deep formation’ without the need for additional delay agents. It complements the near-well rapid plugging function of polyurethane systems, providing a reliable temperature-responsive mechanism and technical basis for the comprehensive management of water channeling in low-permeability, high-temperature, and heterogeneous reservoirs in the Xinjiang Oilfield.

### 2.3. Salt Resistance of PU Plugging System and UF Plugging System

The salt tolerance performance of the two gel systems is shown in Table 4. It can be observed from the table that an increase in the formation water mineralization degree significantly promotes the gelation of PU plugging systems. At 5 g/L and 10 g/L, no gelation occurs. At 20–40 g/L, gelation completes within 1 h. At 60 g/L, gelation time shortens to 0.5 h. For the UF plugging system, increased mineralization delays gelation: at ≤30 g/L, gelation time is 1 h; at 40–60 g/L, it extends to 1.5 h. These opposite responses arise from different crosslinking mechanisms: high salinity reduces the reaction energy barrier for polyurethane but interferes with the condensation polymerization of urea-formaldehyde.

Moreover, high salinity (high ionic strength) reduces the polarity of the aqueous medium, effectively lowering the dielectric constant and thus decreasing the activation energy for the isocyanate-water reaction [23,24]. Additionally, salt ions may disrupt hydrogen-bonded water networks, making water molecules more available for nucleophilic attack on –NCO groups. Consequently, gelation time decreases from 1 h at 20–40 g/L to 0.5 h at 60 g/L. For the urea-formaldehyde system, the curing process involves acid-catalyzed condensation polymerization between methylol urea groups to form methylene (–CH_2_–) and ether (–CH_2_–O–CH_2_–) bridges. High salinity screens electrostatic interactions and reduces the activity of the acid catalyst, thereby retarding the condensation rate. Otherwise, sodium and chloride ions may coordinate with polar hydroxyl and amide groups, stabilizing intermediate species and shifting the equilibrium away from crosslinked products. As a result, gelation time prolongs from 1 h at ≤30 g/L to 1.5 h at 40–60 g/L. In high-salinity environments, salt ions (such as Na^+^, Cl^−^, Ca^2+^, etc.) displace water molecules that were originally adsorbed on the surfaces of polyurethane prepolymers and polyols, which are used to form a hydration film, thereby forming a stable hydrated shell layer. Without the protective hydration film on their surfaces, the inherent water-insolubility of polyurethane reactants is manifested, leading to a decrease in solubility and precipitation. At this point, salt ions neutralize the surface charges of polyurethane colloid particles, reducing electrostatic repulsion, promoting the aggregation of reactants, and forming high-concentration microregions locally, thereby increasing the collision frequency between –NCO and –OH groups [25]. Concurrently, the enrichment of reactants also promotes the cross-linking reactions of polyfunctional isocyanates, accelerating gelation and shortening curing time.

The curing of urea-formaldehyde systems primarily relies on hydrogen ions under acidic conditions for catalysis [26]. First, high salinity increases ionic strength, reduces the activity of effective hydrogen ions, weakens the catalytic effect, and slows down curing. Second, high concentrations of chloride ions generate a common ion effect with chloride ions in ammonium chloride curing agents, inhibiting the hydrolysis of ammonium chloride to produce hydrogen ions, thereby further reducing catalytic capacity [27]. Third, the essence of urea-formaldehyde curing is a dehydration condensation reaction; however, salt ions lock up free water in large quantities, reduce water activity, hinder the forward reaction of urea-formaldehyde dehydration condensation, and decrease contact between reactants and hydrogen ions. Fourth, salt ions can also adsorb onto the surface of urea-formaldehyde colloid particles, shielding active sites of urea-formaldehyde, thickening the colloidal hydration layer to impede particle aggregation, and consuming part of the active groups.

### 2.4. Rheological Property

**Figure 5:**
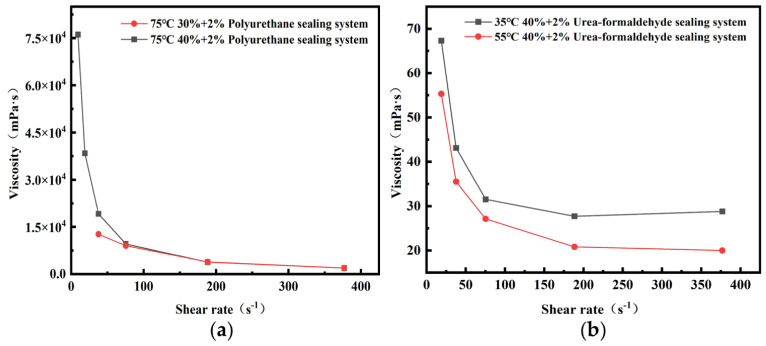
Effect of shear rate on viscosity in (**a**) polyurethane and (**b**) UF plugging systems.

The shear rate on viscosity is shown in Figure 5, both polyurethane and UF plugging systems exhibit shear-thinning behavior within the shear rate range of 0 to 400 s^−1^. As the shear rate increases, the viscosities of the 30% + 2% and 40% + 2% polyurethane systems initially decrease rapidly and then gradually level off. At low shear rates, the initial viscosity of the 40% + 2% system is significantly higher than that of the 30% + 2% system, indicating that higher resin content increases initial viscosity. Above 100 s^−1^, the viscosity difference diminishes. When shear rate exceeds 200 s^−1^, viscosity stabilizes, showing good shear adaptability. Temperature elevation reduces system viscosity and flow resistance. For urea-formaldehyde, a similar shear-thinning trend is observed, with higher temperature lowering viscosity. This behavior ensures easy injection and rapid structural recovery after placement, meeting oilfield requirements.

**Figure 6:**
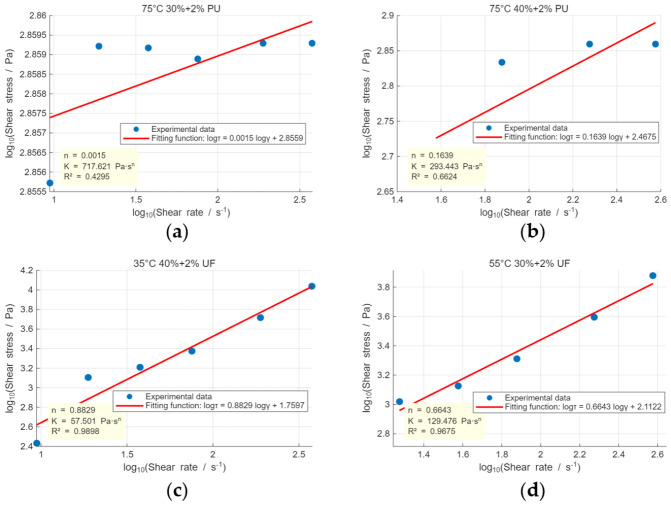
Scatter plot of log(γ) and log(τ) fitting for polyurethane and UF plugging systems. (**a**) 75 °C 30% + 2% PU plugging system; (**b**) 75 °C 40% + 2% PU plugging system; (**c**) 35 °C 40% + 2% UF plugging system; (**d**) 55 °C 40% + 2% UF plugging system.

To quantitatively characterize the shear-thinning behavior, the viscosity–shear rate data were fitted to the power-law model. For the 30% + 2% polyurethane system at 75 °C, the fitted parameters are K = 717.6 $\mathrm{Pa}\cdot s^{n}$ and n = 0.0015 ($R^{2}$ = 1). The extremely low n value (far below 1) indicates an exceptionally strong shear-thinning behavior, which is consistent with the rapid viscosity drop observed in Figure 5. For the 40% + 2% urea-formaldehyde system at 35 °C, the parameters are $K=57.5\;\mathrm{Pa}\cdot s^{n}$ and n=0.8829 ($R^{2}=0.9898$), indicating a near-Newtonian behavior (n close to 1) with only slight shear thinning. At 55 °C, the UF system exhibits more pronounced shear thinning with $K=1.295\times 10^{5}\;\mathrm{Pa}\cdot s^{n}$ and n=0.6643 ($R^{2}=0.9675$). The increase in temperature reduces the flow behavior index and dramatically increases the consistency coefficient, reflecting temperature-triggered thickening that is crucial for deep fluid diversion. The PU system, despite its poor power-law fit ($R^{2}=0.4295$), shows excellent injectability under high shear rates due to its extremely low viscosity at elevated shear. The fitted data plot is shown in Figure 6. All fitting results are summarized in Table 5, Table 6, Table 7, Table 8 and Table 9.

(1) $$\tau =K\cdot \gamma^{n}$$

Equation (1) describes a power-law model. It is used to describe the relationship between shear stress and shear rate in non-Newtonian fluids. The τ is the shear stress (Pa), γ the shear rate (s^−1^), K is the consistency coefficient ($\mathrm{Pa}\cdot s^{n}$), and n is the flow behavior index (dimensionless).

(2) τ=η×γ

Equation (2) describes the rheological equation of a Newtonian fluid. It is used to describe the linear relationship between internal shear stress and shear rate of a Newtonian fluid under laminar flow conditions, where viscosity η serves as an indicator of the fluid’s resistance to shear deformation.

(3) $$\;log\left(K\right)\approx log\left(\tau \right)\;-\;n\cdot log\left(\gamma \right)\;$$

Equation (3) describes the calculation method of the consistency coefficient K.

### 2.5. Compressive Strength

**Figure 7:**
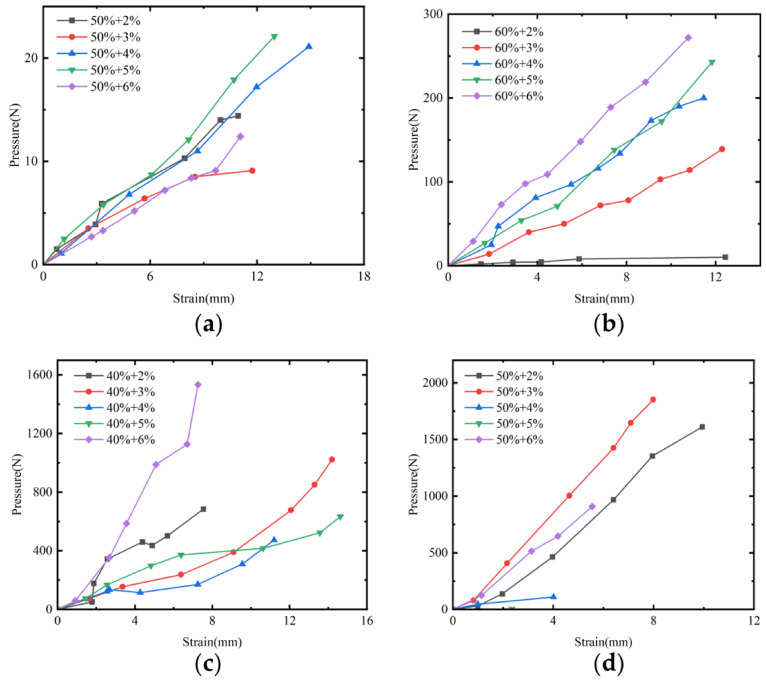
Stress–strain curves of different polyurethane and urea-formaldehyde resin plugging systems. (**a**) 50% polyurethane resin concentration with different curing agent concentrations; (**b**) 60% polyurethane resin concentration with different curing agent concentrations; (**c**) 40% urea-formaldehyde resin concentration with different curing agent concentrations; (**d**) 50% urea-formaldehyde resin concentration with different curing agent concentrations.

The compressive strength of polyurethane resin grouting agent and urea-formaldehyde resin grouting agent was tested using digital force gauges, and the results are shown in Figure 7. As can be seen from Figure 7a,b, the compressive strength of the polyurethane plugging system continuously increases with the elevation of resin and curing agent concentrations. The compressive strength of the 60% polyurethane resin system is approximately 10 times that of the 50% system. Among them, the 60% + 6% formulation achieves a compressive strength of 272 N (854 Pa) under a 12 mm strain, whereas the 60% + 2% formulation is only 10.2 N (32 Pa). The polyurethane system constructs a cross-linked network through flexible urethane bonds, allowing molecular chains to freely bend and extend, thereby possessing good toughness and deformation capacity. However, due to the inherent insufficient rigidity of its own skeleton, its overall hardness and compressive performance are significantly weaker than those of the urea-formaldehyde system.

From the test results of the urea-formaldehyde resin plugging system shown in Figure 7c,d, it can be observed that there are significant differences in mechanical properties among different formulations. The 50% + 3% formulation exhibits the optimal compressive performance, reaching a compressive strength of 1852 N (5815 Pa) under an 8 mm strain, with a peak value approaching 2000 N (6280 Pa). The 40% + 6% formulation achieves a compressive strength of 1533 N (4813 Pa) under a 7.25 mm strain, which is much higher than the 171 N (537 Pa) of the 40% + 4% formulation at the same resin concentration. This fully demonstrates the regulatory effect of curing agent dosage on the mechanical properties of the material. The reason lies in the fact that after curing, urea-formaldehyde can form a structurally stable, rigid three-dimensional network of triazine rings. The fixed triazine ring structure and difficulty in molecular chain torsion and slippage make the material less prone to deformation under external forces, thus resulting in high overall compressive strength and excellent structural stability. Overall comparison clearly shows that the urea-formaldehyde plugging system has significantly better compressive performance than the polyurethane system.

Recent studies have reported that a high-temperature-resistant gelled resin plugging system based on urea-formaldehyde resin achieved a compressive strength of 9.3 MPa at 100–140 °C, and the optimized formulation exhibited a bearing pressure of 13.95 MPa in wedge-shaped fractures at 140 °C [28]. Similarly, a renewable epoxy soybean oil-based resin plugging agent (BEOPA) developed for damage repair achieved an impressive compressive strength of 93.7 MPa with breakthrough pressures exceeding 29.7 MPa in 1 mm crack cores [29]. Zhao et al. [30] developed a multistage enhanced viscoelastic gel with a maximum compressive strength of 545.2 kPa and demonstrated excellent water shutoff performance with breakthrough pressures up to 9 MPa. Chen et al. [31] synthesized a double-network hydrogel reinforced with acrylic-grafted nanocellulose, achieving a maximum compressive strength of 2.5 MPa at 90% strain, with breakthrough pressure gradients reaching 4.9–104.63 MPa/m in sand-pack plugging tests. Wang et al. [32] developed a high-strength and self-degradable sodium alginate/polyacrylamide preformed particle gel (d_PPG) with a storage modulus of 86,445 Pa, which is nearly 20 times higher than conventional PPGs, and demonstrated a plugging efficiency of 99.83% on open fractures.

**Figure 8:**
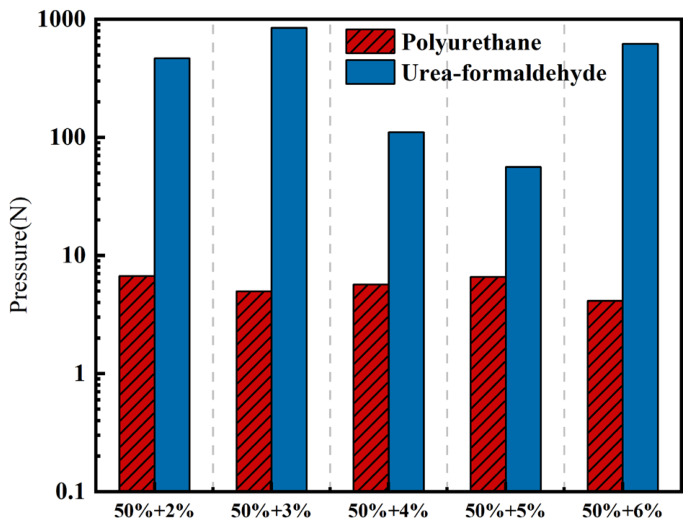
Compressive strength of different samples at the same displacement (4 mm).

As shown in Figure 8, for the PU plugging system, the compressive strength increases gradually with the elevation of resin concentration and curing agent concentration. Among these, the 60% + 6% PU plugging system exhibits the highest strength, yet it remains significantly lower than the minimum UF plugging system. For the UF plugging system, the compressive strength is highly sensitive to resin concentration: the strength at 50% resin concentration (600–1300 N) is markedly higher than that at 40% resin concentration (171–200 N). At the same resin concentration, increasing the curing agent concentration can more than double the strength.

The PU plugging system constructs a cross-linked network via flexible urethane bonds, allowing molecular chains to freely bend and extend, thereby exhibiting good toughness and deformation capacity. However, its insufficient skeletal rigidity results in relatively low compressive strength. In contrast, the UF plugging system forms a stable, rigid three-dimensional network of triazine rings upon curing, where molecular chain torsion and slip are difficult. This makes deformation less likely under external forces, leading to significantly higher compressive strength. This structural difference is quantitatively validated in Figure 8.

From this, we can see that the UF plugging system is suitable for high-stress plugging in the near-wellbore area, such as regions near the wellbore that need to withstand high pressure differentials and prevent water channeling. However, it has a narrow construction window and requires precise control of the formulation and mixing time. Different from the UF plugging system, the PU plugging system is applicable for deep reservoir displacement and adjustment due to its moderate strength, good toughness, and insensitivity to fluctuations in curing agent concentration, which prevents premature brittle fracture and failure during deep migration.

Figure 8 clearly reveals the mechanical performance complementarity of the two systems: UF provides ultra-high strength but has a narrow construction window, while PU offers good toughness and a wide operational window. In practical applications, the appropriate selection or combination should be made based on the plugging location and pressure grade.

### 2.6. Microstructure

**Figure 9:**
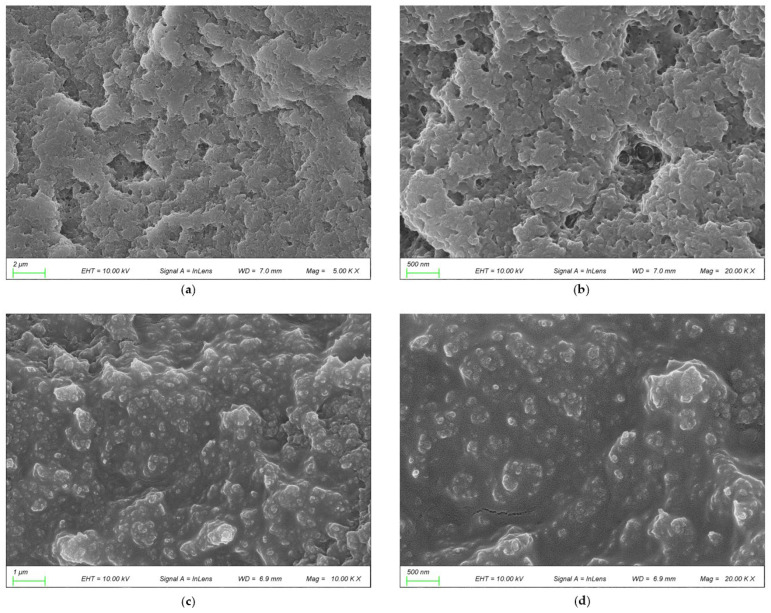
Scanning electron microscope image. (**a**) PU plugging system, 2 μm; (**b**) PU plugging system, 500 nm; (**c**) UF plugging system, 1 μm; (**d**) UF plugging system, 500 nm.

Scanning electron microscopy (SEM) images are shown in Figure 9. Figure 7b and Figure 9a exhibit the microstructure of the cured polyurethane plugging system, which presents a dense, continuous, and pore-free morphology. At low magnification (Figure 9a, scale bar 1 μm), the system overall displays a uniform blocky continuous phase with no obvious voids or cracks; high-magnification observation (Figure 9b, scale bar 500 nm) further reveals its tightly cross-linked internal morphology, where molecular chains are uniformly entangled to form a dense three-dimensional network. This highly cross-linked pore-free structure directly endows the system with excellent mechanical strength and plugging performance, serving as the key structural basis for achieving high-strength plugging in the near-wellbore region.

In stark contrast, Figure 9c,d display the microstructure of the cured urea-formaldehyde resin plugging system, which exhibits a uniformly distributed porous network. At low magnification (Figure 9c, scale bar 2 μm), a large number of uniformly distributed, moderately sized pores within the system can be clearly observed; high-magnification observation (Figure 9d, scale bar 500 nm) further demonstrates its three-dimensional interconnected network composed of alternating resin matrix and pores. This porous structure provides fluid flow channels, enabling the system to possess good permeability adaptability and offering the necessary structural conditions for achieving controllable fluid flow diversion in depth.

### 2.7. Plugging Performance

#### 2.7.1. Blockage Rate and Penetration Rate

**Figure 10:**
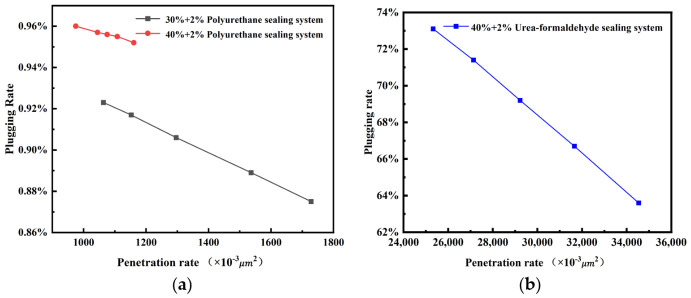
Penetration rate and blockage rate of (**a**) polyurethane and (**b**) UF plugging systems.

Plugging tests for polyurethane and urea-formaldehyde were conducted using the forward and reverse displacement method, with the results shown in Figure 9. Figure 10 indicates that the PU plugging system exhibits superior plugging performance in the pore structure of the reservoir formation in a certain block of an oilfield in Xinjiang compared to the UF plugging system. Moreover, the plugging performance increases with the increase in resin concentration. For the 30% + 2% PU plugging system, the maximum plugging efficiency can reach 92.5%, and for the 40% + 2% PU plugging system, it can reach as high as 96.0%, demonstrating the strengthening effect of higher resin concentration on plugging stability. However, the UF plugging system can only achieve a maximum plugging efficiency of 73%, and its efficiency decreases more significantly with increasing penetration. This suggests that the UF plugging system has lower compatibility with the pore structure of the reservoir formation in this block compared to the PU plugging system. The high-penetration formations in a certain block of the Xinjiang Oilfield have wide pore channels, resulting in smaller fluid flow resistance. This makes it more difficult for the UF plugging system to remain in the pores and undergo cross-linking gelation, leading to a decrease in plugging efficiency as penetration increases. In contrast, the PU plugging system, due to its higher resin concentration and denser cross-linked network, can better adapt to changes in formation pores, thereby maintaining a more stable plugging efficiency and being more suitable for the channel plugging requirements of this high-penetration formation.

#### 2.7.2. Breakthrough Pressure and Penetration Rate

Penetration rate is a key criterion for screening and evaluating plugging agents. By analyzing plugging efficiency data, targeted adjustments can be made to plugging agent formulations, dosages, or injection parameters.

**Figure 11:**
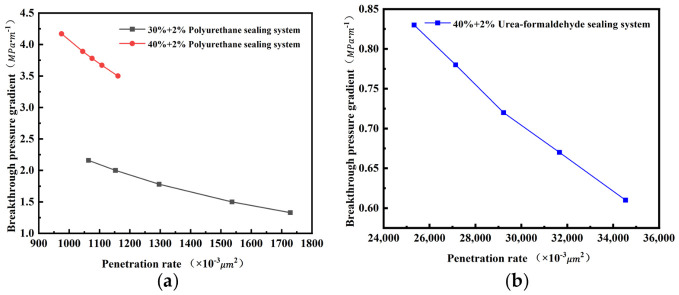
Penetration rate and breakthrough pressure gradient of (**a**) polyurethane and (**b**) UF plugging systems.

Figure 11 shows that as the penetration rate increases, for the 30% + 2% PU plugging system, the breakthrough pressure gradient decreases by approximately 0.9 MPa·m^−1^; for the 40% + 2% PU plugging system, the decrease is about 0.7 MPa·m^−1^. The 40% + 2% overall level is significantly higher than that of the 30% + 2% system, which the highest parameter could reach at 4.2 MPa·m^−1^ (the corresponding permeability is 4500 μm^2^), demonstrating the strengthening effect of higher resin concentration on anti-breakthrough performance. The UF plugging system has lower anti-breakthrough capability than the PU plugging system. For the UF plugging system, the breakthrough pressure gradient decreases by approximately 0.22 MPa·m^−1^ with increasing formation penetration, a decline magnitude significantly greater than that of the PU plugging system. Compared to the PU plugging system, the UF plugging system has a lower initial breakthrough pressure gradient and a less steep downward trend with increasing penetration. This indicates that the UF plugging system has weaker cross-linking within pores, leading to easier fluid breakthrough through the plugging layer. This further confirms that, compared to the UF plugging system, the PU plugging system is more suitable for plugging in this oilfield block.

## 3. Conclusions

Addressing the severe water channeling issues prevalent in the low-permeability, high-temperature, and highly heterogeneous reservoirs of the Xinjiang Oilfield, this study systematically investigated two advanced chemical plugging systems: the PU plugging system and the UF plugging system. Under simulated reservoir conditions, both systems were optimized and evaluated to provide a technical solution for conformance control in challenging environments.

The study comprehensively analyzed the gelation mechanisms, rheological properties, compressive strength, salt resistance, and microstructural characteristics of both systems. Data indicated that the PU system exhibits rapid gelation primarily controlled by resin concentration, achieving a high plugging efficiency of 96% and a breakthrough pressure gradient of 4.2 MPa·m^−1^, making it ideal for high-strength near-wellbore plugging. In contrast, the UF system demonstrated a distinct temperature-triggered gelation behavior (activated above 60 °C) and superior compressive strength (up to 1852 N), coupled with a porous microstructure that facilitates deep fluid diversion. Notably, the two systems displayed opposite responses to salinity: high mineralization conditions accelerated the PU gelation but delayed the UF gelation.

These experimental findings hold significant practical value for the development of the Junggar Basin, offering a dual-system strategy that leverages the complementary strengths of PU and UF resins. Looking forward, research will focus on the synergistic application of these two systems to achieve “deep profile control combined with near-wellbore sealing.” Future field pilots will aim to validate the long-term stability and economic feasibility of this combined approach in high-temperature, high-salinity reservoirs, thereby providing a robust technical foundation for enhancing oil recovery in similar complex formations.

## 4. Materials and Methods

### 4.1. Material

PU plugging system resin solution, solid content 35%, model YC-312, and PU plugging system curing agent, solid content 100%, model YC-8100, were purchased from Anhui Yuanchen New Material Co., Ltd. (Bengbu, China). UF plugging system resin, solid content 50%, industrial grade, and acid-based curing catalyst were obtained from Shanghai Sinopharm Chemical Reagent Co., Ltd. (Shanghai, China). 100-mesh fine sand resembling table salt and 120-mesh fine sand were purchased from Jinhong Heli Colored Sand Factory. The water used in the gel system configuration is simulated formation water. The ion content of the formation water is based on the data from the Second Oil Production Plant of Xinjiang. In accordance with the literature, the formula of simulated formation water for the Second Oil Production Plant of a certain oilfield in Xinjiang is shown in Table 10. The total dissolved solids (TDS) of formulation water is 30,000 mg/L. Simulated formation water with different mineralization degrees was prepared using analytical-grade salts and deionized water.

### 4.2. Experimental Instruments

A beaker, dropper, stirrer, volumetric flask (1000 mL), electronic balance, glass bottle (30 mL), and other regular experimental instruments were used. The constant temperature water bath is built by Yiheng (Shanghai, China). The vacuum drying oven is manufactured by Yiheng (Shanghai, China). The NDJ-8S viscometer is manufactured by Yueping (Shanghai, China). The HBCD-70 compressive strength tester is built by Huabao (Yangzhou, China). The digital dynamometer is built by Shenzhen Ailigu Instrument Co., Ltd. (Shenzhen, China). The sand-packed tube is manufactured by Chengyue Experimental Instrument Equipment Factory (Zhengzhou, China), with dimensions of 25 × 150 mm.

### 4.3. Preparation of Gel Systems

The following steps are the preparation of the gel systems:

1. Add a certain amount of simulated formation water.
2. Add a certain amount of PU plugging system or UF plugging system resin, at 800 r/min, and stir for 30 min until the mixture is homogeneous.
3. Add a certain amount of curing agent, at 800 r/min, and stir for 30 min until the mixture is homogeneous.
4. Pour the prepared gel system into a 30 mL observation bottle, and then place it in an 80 °C oven for continuous observation for 4 h.

### 4.4. Methods

#### 4.4.1. Fourier Transform Infrared Spectroscopy (FTIR)

FTIR is performed using the KBr pellet method. Samples are dried in a vacuum oven at 120 °C for 1 h, ground into fine powder, mixed with dried KBr powder (1:100 ratio), and pressed into transparent pellets. Spectra are collected over the range of 400–4000 cm^−1^ with 32 scans at 4 cm^−1^ resolution.

#### 4.4.2. SEM

SEM samples are prepared by freeze-fracturing cured specimens in liquid nitrogen, sputter-coating with gold for 90 s, and imaging at various magnifications with an accelerating voltage of 5–10 kV.

#### 4.4.3. Gelation Time Analysis

The analysis is conducted as follows. Firstly, prepare the polyurethane and urea-formaldehyde plugging system, with the specific preparation process detailed in Section 4.3; Secondly, place the prepared plugging system in an 80 °C oven and observe the curing status every half hour until the sample, when tilted 45°, does not flow within 3 s.

#### 4.4.4. Rheological Measurements

Viscosity measurements are conducted using the NDJ-8S rotational viscometer, manufactured by Shanghai Youyi Instrument Co., Ltd. (Shanghai, China) according to the GB/T 22235-2008 standard [33]. Samples are placed in the measurement cup and equilibrated at the target temperature for 15 min before testing. Shear rate sweeps are performed from 1 to 100 s^−1^, and viscosity is recorded at each shear rate. For temperature-dependent studies, viscosity was monitored at a constant shear rate of 10 s^−1^, while the temperature increased from 35 °C to 80 °C at a rate of 1 °C/min.

#### 4.4.5. Compressive Strength Testing

**Figure 12:**
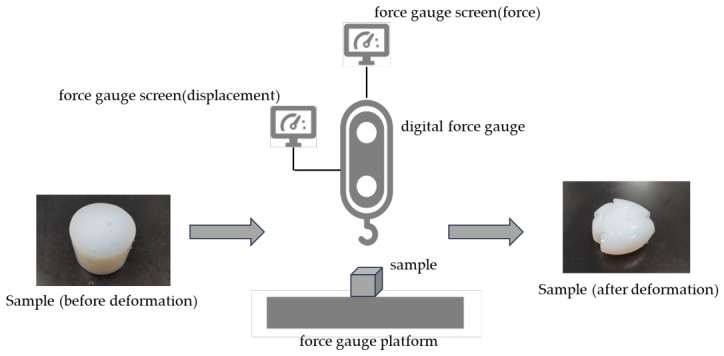
Flowchart of compressive strength test.

The device diagram is shown in Figure 12. Take a solidified block of sealant with a radius of 1 cm and a thickness of 2 cm, place it on a digital force gauge for strength testing. Its compressive strength is determined when significant deformation occurs.

(4) $$\sigma =\frac{F}{A}$$

Equation (4) describes compressive strength. It is the maximum stress that a material can withstand when subjected to pressure until failure under conditions of no lateral constraint. The σ represents the compressive strength, whose unit is N/m^2^. The *F* represents the force which is applied to the cross-section of a material, whose unit is N. The *A* is the cross-sectional area of the sample, whose unit is m^2^.

#### 4.4.6. Plugging Performance Evaluation

**Figure 13:**
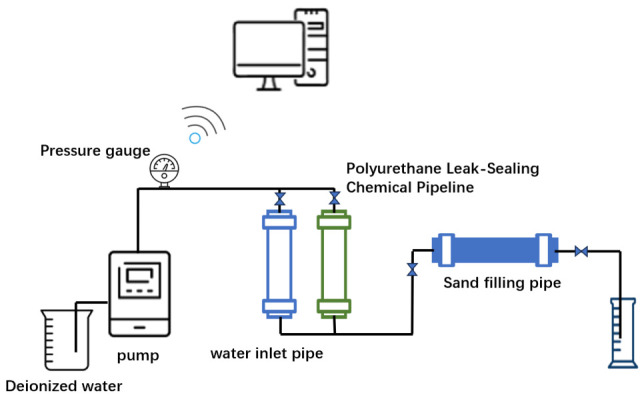
Plugging displacement device.

A plugging displacement device, as shown in Figure 13, is used for plugging evaluation: Fill the sand-packed tube with sand and compact it to simulate the reservoir, and determine the dry weight of the sand. Add simulated formation water to submerge the sand-packed tube, determine the wet weight of the sand through vacuuming, and calculate the pore volume and porosity. Use the displacement device to inject formation water into the sand-packed tube in the forward direction at a flow rate of 1 mL/min. After the injection pressure is stable and the volume of injected formation water exceeds 2 pore volumes (PV), record the pressure and calculate the initial water-phase penetration. Inject the PU plugging system channeling plugging system into the sand-packed tube in the forward direction until reaching 1 PV, then place it in an oven at 85 °C for aging for 2 h. Cool to room temperature, inject simulated formation water in the reverse direction for a water-phase reverse displacement experiment, continuously record the pressure change until the reverse displacement water volume reaches 10 PV, and calculate the water-phase penetration after plugging, water-phase plugging rate, and breakthrough pressure. The equations of penetration rate, plugging rate, and breakthrough pressure are shown below.

(5) $$K=\frac{Q\mu L}{A\Delta P}$$

Equation (5) describes penetration. It is a measure of how easily a fluid can flow through a porous material, such as rock or soil. The capital K represents penetration, the unit of which is $m^{2}$; Q denotes the flow rate of the fluid pumped through the sand-filled tube, the unit of which is $m^{3}/s$; μ signifies the fluid viscosity, the unit of which is Pa·s; L corresponds to the length of the sand-packed tube, the unit of which is m; A indicates the cross-sectional area of the tube, the unit of which is $m^{2}$; ∆P designates the pressure differential, the unit of which is Pa.

(6) $$P_{L}=\frac{P_{\max}}{L}$$

Equation (6) describes the breakthrough pressure gradient. It is the change in pressure per unit distance that drives fluid movement in porous media. The capitalized $P_{max}$ represents the breakthrough pressure after the simulated core is blocked in the sand filling pipe, the unit of which is Pa. The capitalized L represents the length of the sand filling pipe, the unit of which is m. $P_{L}$ represents the breakthrough pressure gradient, the unit of which is Pa.

(7) $$F_{s}=\frac{K_{1}-K_{2}}{K_{1}}\times 100\%$$

Equation (7) describes the plugging rate. It is the percentage ratio of the blocked pore volume to the original pore volume, showing how well a plugging agent reduces penetration. The $F_{S}$ represents the plugging rate; the $K_{1}$ represents the water injection penetration before the plugging system seals the simulated core, the unit of which is mD;${\;K}_{2}$ represents the water injection penetration after the plugging system seals the simulated core, the unit of which is mD.

## Figures and Tables

**Table 1:** Resin concentration and curing agent concentration on gelation status of polyurethane channel plugging system.

Curing Agent Concentration	Resin Concentration				
	20%	30%	40%	50%	60%
0.5%	4.5 h	1 h	1 h	1.5 h	1.5 h
1.5%	4.5 h	1 h	1 h	1.5 h	1.5 h
2%	4.5 h	1 h	1 h	1.5 h	1.5 h
2.5%	4.5 h	1 h	1 h	1.5 h	1.5 h
3%	4.5 h	1 h	1 h	1.5 h	1.5 h

**Table 2:** Resin concentration and curing agent concentration on gelation status of urea-formaldehyde channel plugging system.

Curing Agent Concentration	Resin Concentration				
	20%	30%	40%	50%	60%
2%	3.5 h	3 h	2 h	1.5 h	1.5 h
3%	3 h	3 h	2 h	1.5 h	1.5 h
4%	/	3 h	2 h	1.5 h	1.5 h
5%	/	3 h	2 h	1.5 h	1.5 h
6%	/	3 h	2 h	1.5 h	1.5 h

**Table 3:** Gelation time for different plugging system samples.

Number	Recipe	Temperature/°C	Gelation Time/min
1	30% + 2% PU plugging system	50	45
		60	25
		70	5
		80	6
2	40% + 2% PU plugging system	50	35
		60	15
		70	7.5
		80	5
3	40% + 2% UF plugging system	50	-
		60	60
		70	-
		80	-

**Table 4:** Mineralization degree and gelation status of PU and UF plugging system.

Formation Water Mineralization Degree (g/L)	5	10	20	30	40	60
**Gel System**	**Gelation Time (h)**					
30% + 2% PU plugging system	No gelation	No gelation	1 h	1 h	1 h	0.5 h
40% + 2% PU plugging system	No gelation	No gelation	1 h	1 h	1 h	0.5 h
UF plugging system	No gelation	No gelation	1 h	1 h	1 h	0.5 h

**Table 5:** Summary calculation table of 30% + 2% PU data.

75 °C 30% + 2% PU Plugging System		
Shear Rate *γ*/s^−1^	Viscosity η/(mPa·s)	Shear Stress τ/Pa
376.98	1918	723.25
188.49	3836	723.25
75.396	9584	722.59
37.698	19,180	723.05
18.849	38,361	723.13
9.4245	76,102	717.33

**Table 6:** Summary calculation table of 40% + 2% PU data.

75 °C 40% + 2% PU Plugging System		
Shear Rate *γ*/s^−1^	Viscosity η/(mPa·s)	Shear Stress τ/Pa
376.98	1918	723.25
188.49	3837	723.25
75.396	9039	681.51
37.698	12,686	478.25

**Table 7:** Summary calculation table of 40% + 2% UF data.

35 °C 40% + 2% UF Plugging System		
Shear Rate *γ*/s^−1^	Viscosity η/(mPa·s)	Shear Stress τ/Pa
376.98	28.8	10,857.02
188.49	27.7	5221.17
75.396	31.5	2374.97
37.698	43.1	1624.78
18.849	67.3	1268.54
9.4245	28.8	271.43

**Table 8:** Summary calculation table of 30% + 2% UF data.

55 °C 30% + 2% UF Plugging System		
Shear Rate *γ*/s^−1^	Viscosity η/(mPa·s)	Shear Stress τ/Pa
376.98	20	7539.6
188.49	20.8	3920.59
75.396	27.1	2043.23
37.698	35.5	1338.28
18.849	55.3	1042.35

**Table 9:** Table of corresponding calculated coefficients for power-law models of four gel systems.

System	Flow Behavior Index n	Consistency Coefficient K ($\mathrm{Pa}\cdot s^{n}$)	$R^{2}$
30% + 2% Polyurethane 75 °C	0.0015	717.629	0.4295
40% + 2% Polyurethane 75 °C	0.1638	293.562	0.6624
40% + 2% Urea-formaldehyde 35 °C	0.8829	57.501	0.9898
40% + 2% Urea-formaldehyde 55 °C	0.6643	129,476	0.9675

**Table 10:** Formula of formation water in the second oil production plant of Xinjiang Oilfield.

Component	NaCl	NaHCO_3_	CaCl_2_	MgCl_2_	Na_2_SO_4_
content (mg/L)	7568.86	7087.6	23.5	21.4	253.6

## Data Availability

The data presented in this study are openly available in the article.

## Abbreviations

The following abbreviations are used in this manuscript:

PU	Polyurethane
UF	Urea-formaldehyde
PV	Pore volume
TDS	Total dissolved solids
DPV	Pore volume
SEM	Scanning electron microscopy
FTIR	Fourier transform infrared spectroscopy
EOR	Enhanced oil recovery
BEPOA	renewable epoxy soybean oil-based resin plugging agent
d_PPG	self-degradable sodium alginate/polyacrylamide preformed particle gel
PPG	Preformed particle gels
